# Supplementary material for: Strategies and Patterns of Codon Bias in Molluscum Contagiosum Virus
Source: Pathogens. 2021 Dec 20;10(12):1649. doi: 10.3390/pathogens10121649 (PMC8703355; doi:10.3390/pathogens10121649)
Supplement: Supplementary file 1 [file pathogens-10-01649-s001.zip › pathogens-1501935-supplementary.pdf]

MH320547 (MCV 1)

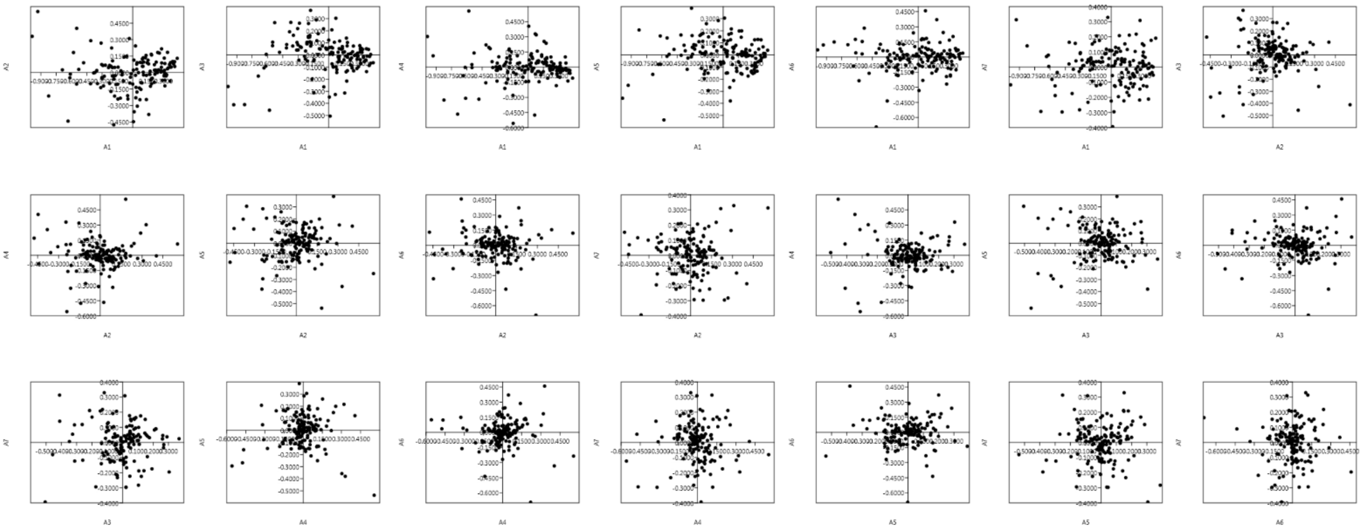

Figure S1. Coding sequences of MH320547.

MH320552 (MCV 1)

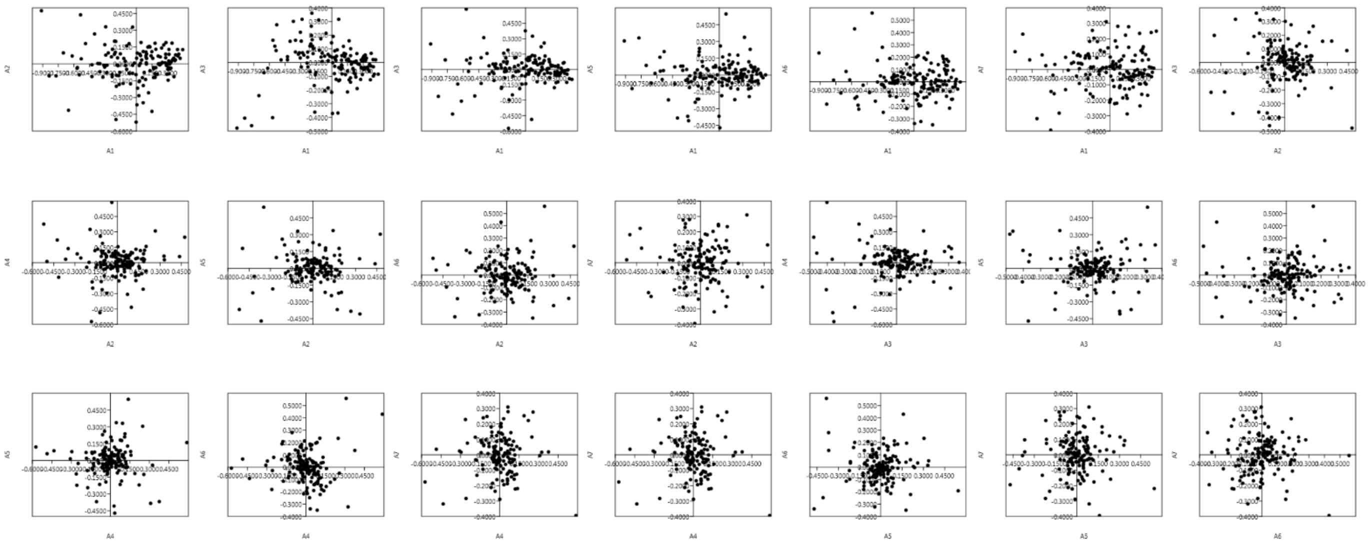

MH320553 (MCV 1)

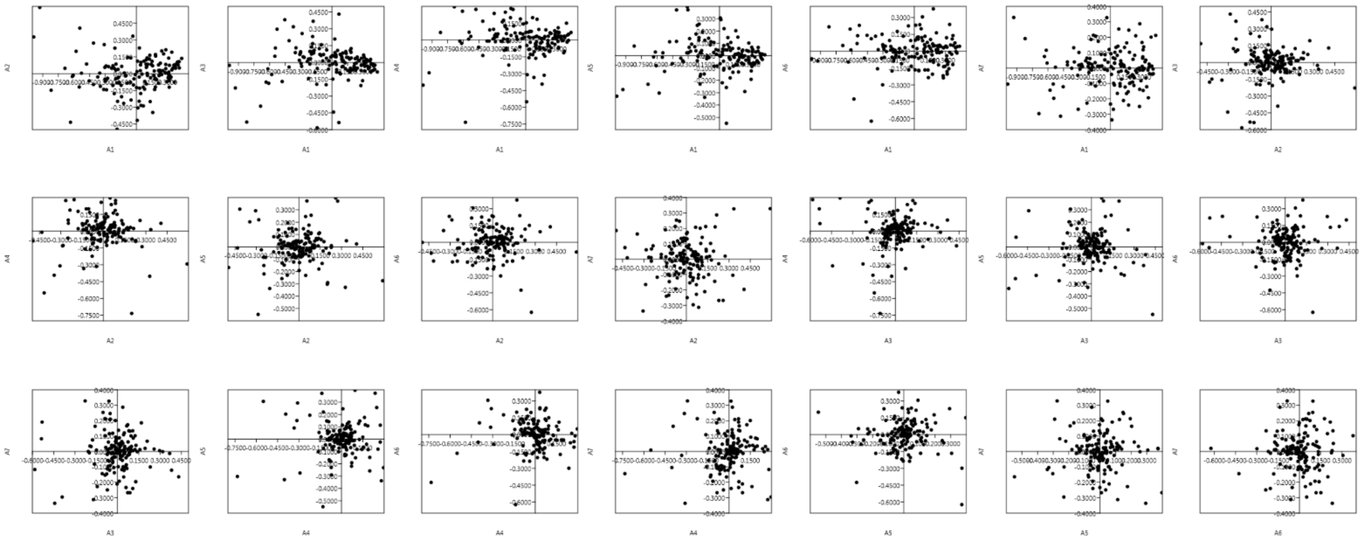

Figure S2. Coding sequences of MH320552 and MH320553.

MH320554 (MCV 1)

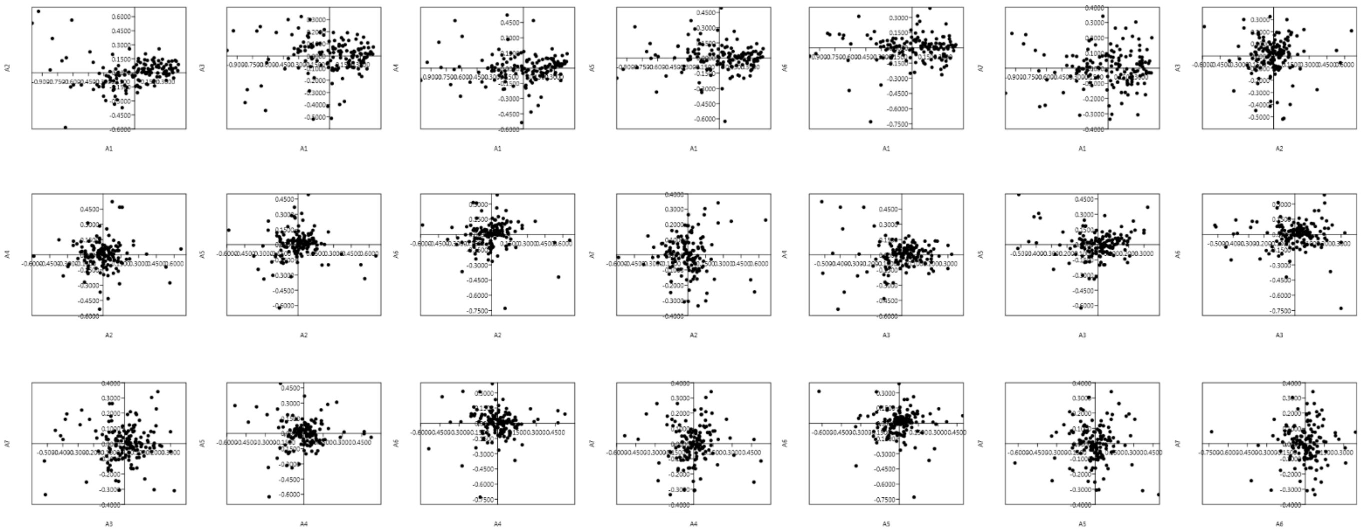

MH320555 (MCV 1)

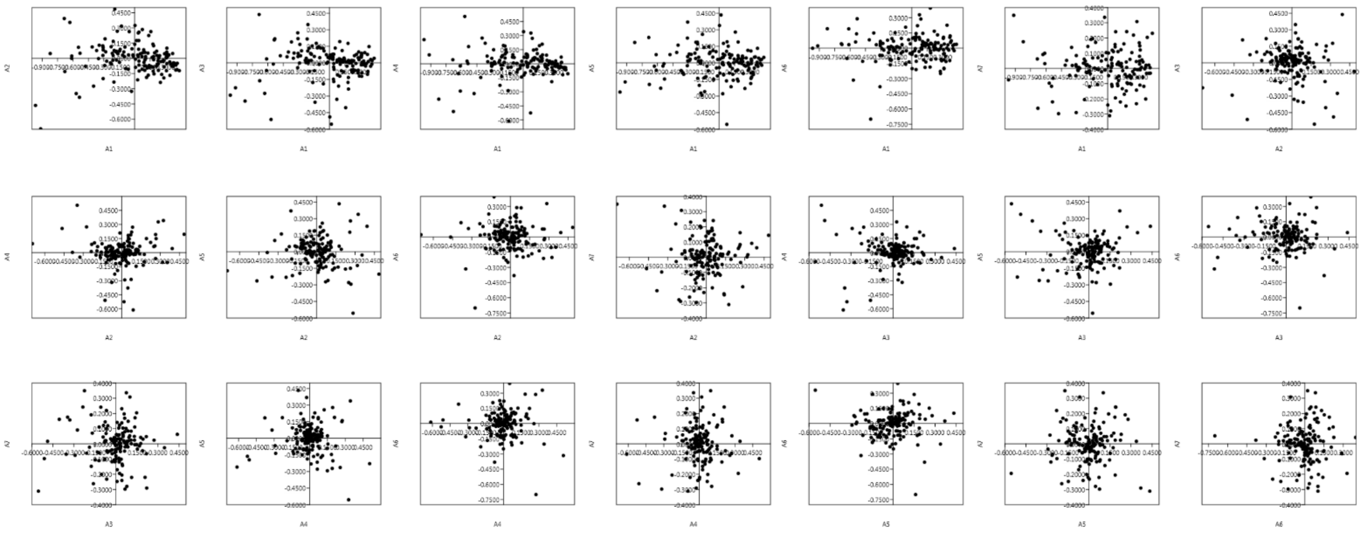

Figure S3. Coding sequences of MH320554 and MH320555.

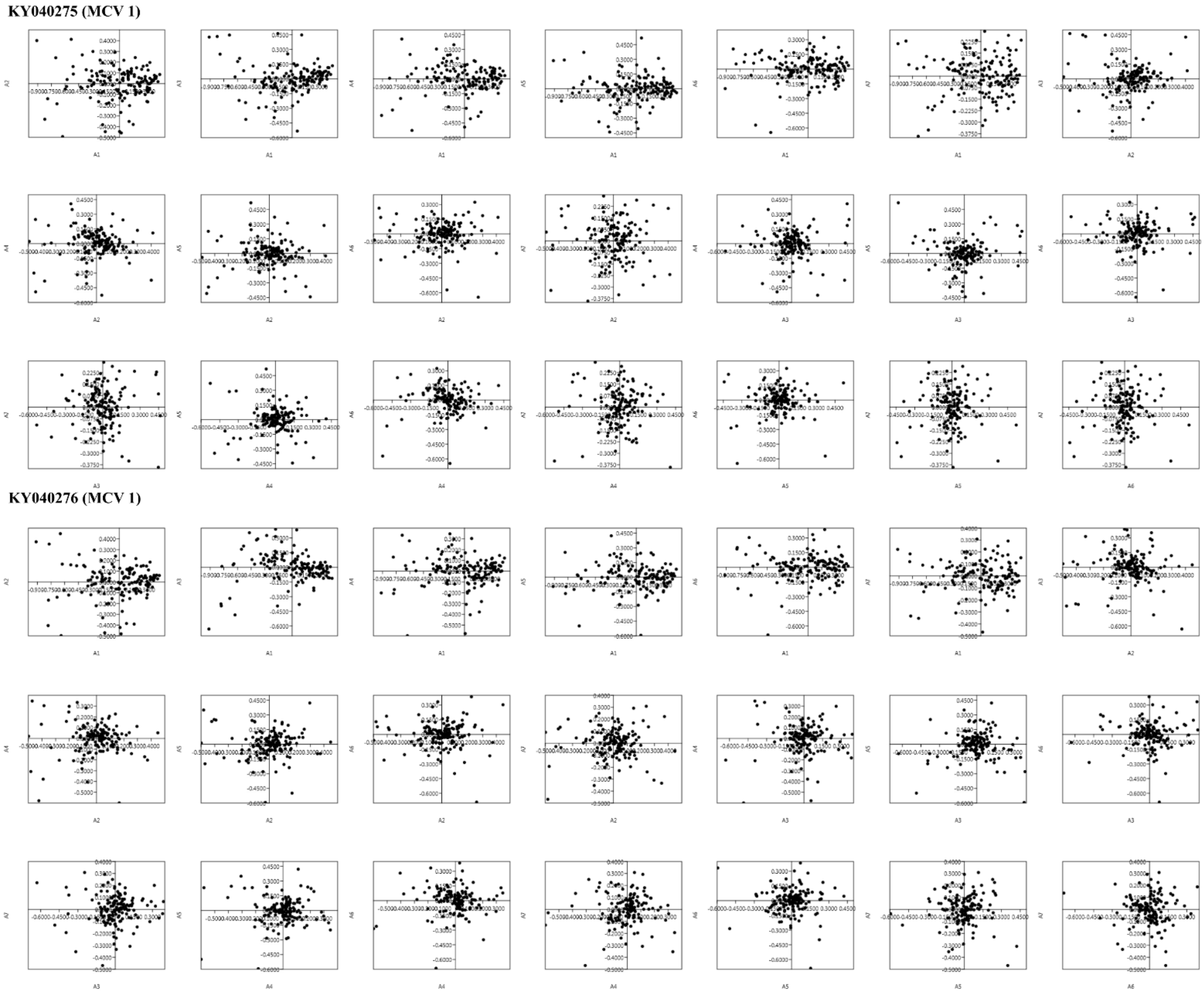

Figure S4. Coding sequences of KY040275 and KY040276.

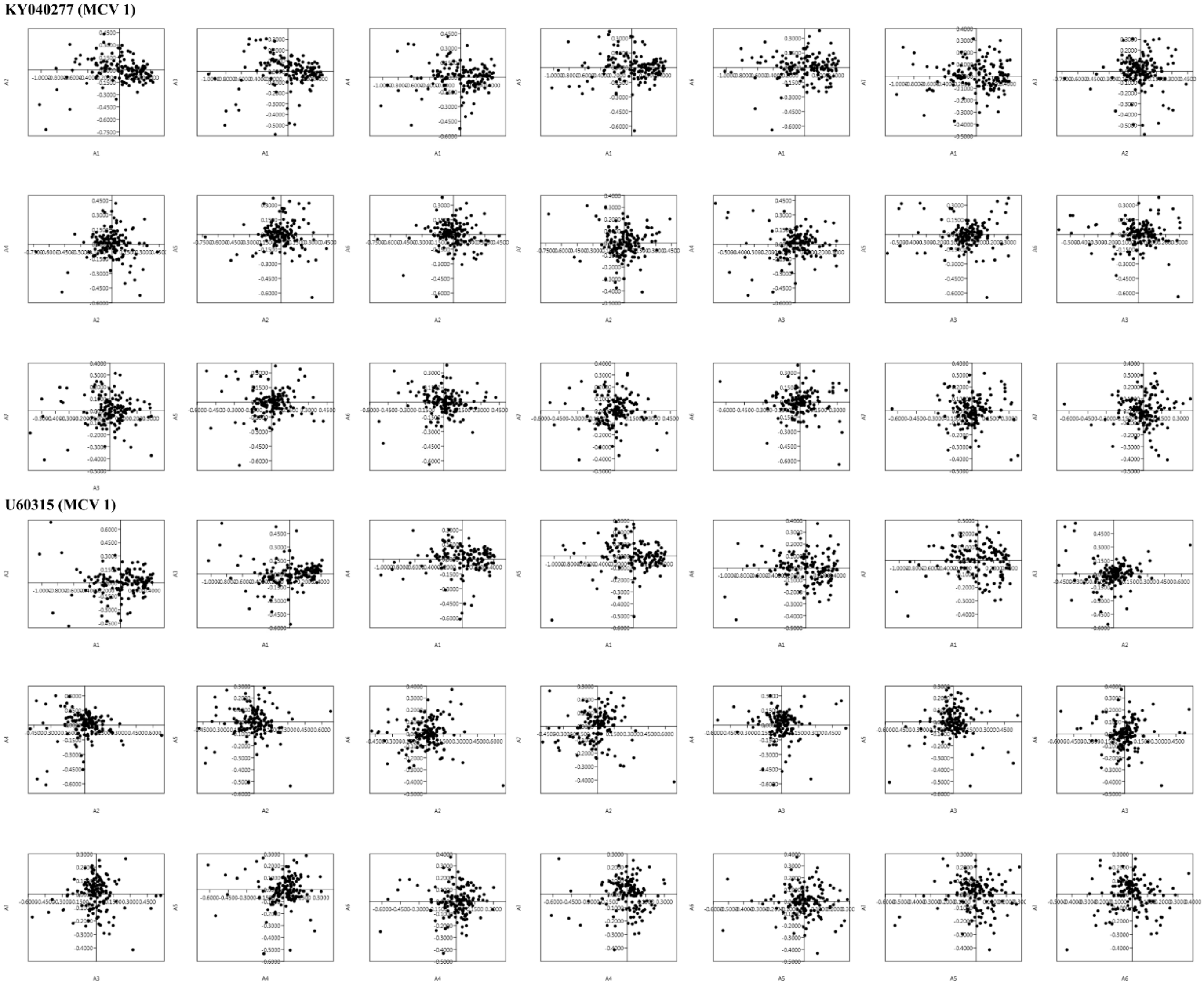

Figure S5. Coding sequences of KY040277 and U60315.

MH320548 (MCV 2)

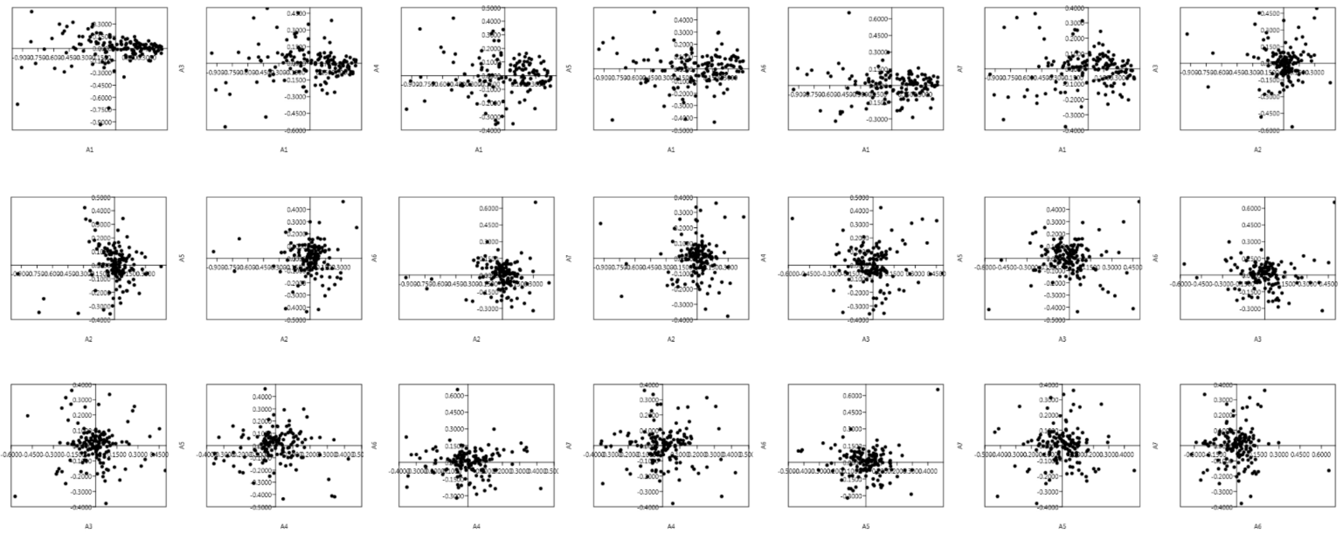

MH320549 (MCV 2)

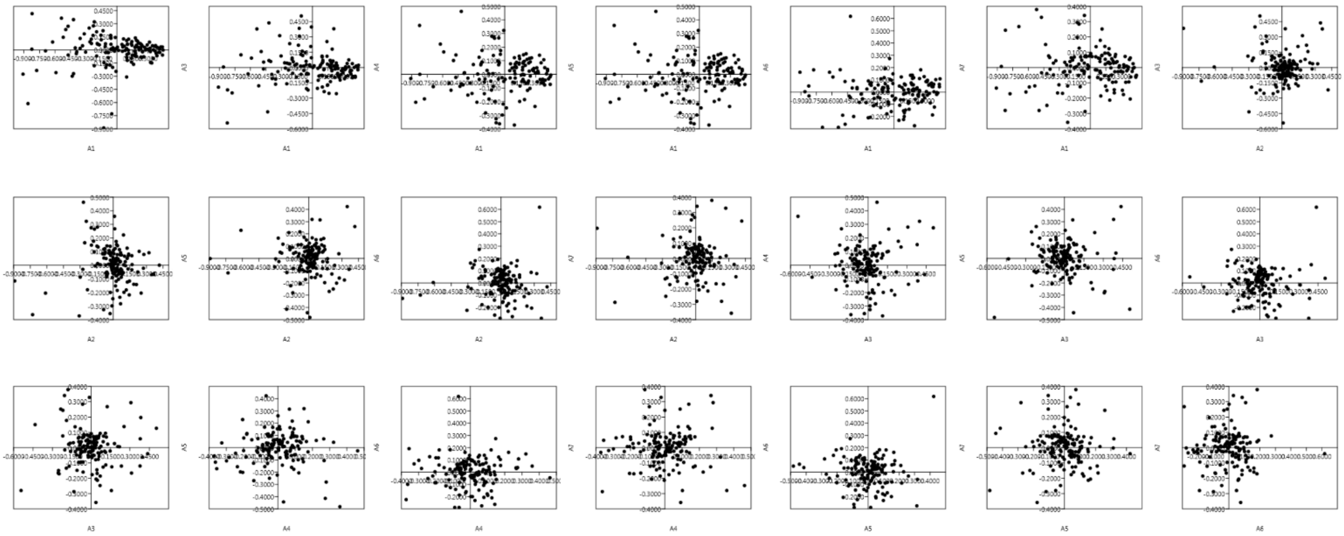

Figure S6. Coding sequences of MH320548 and MH320549.

MH320551 (MCV 2)

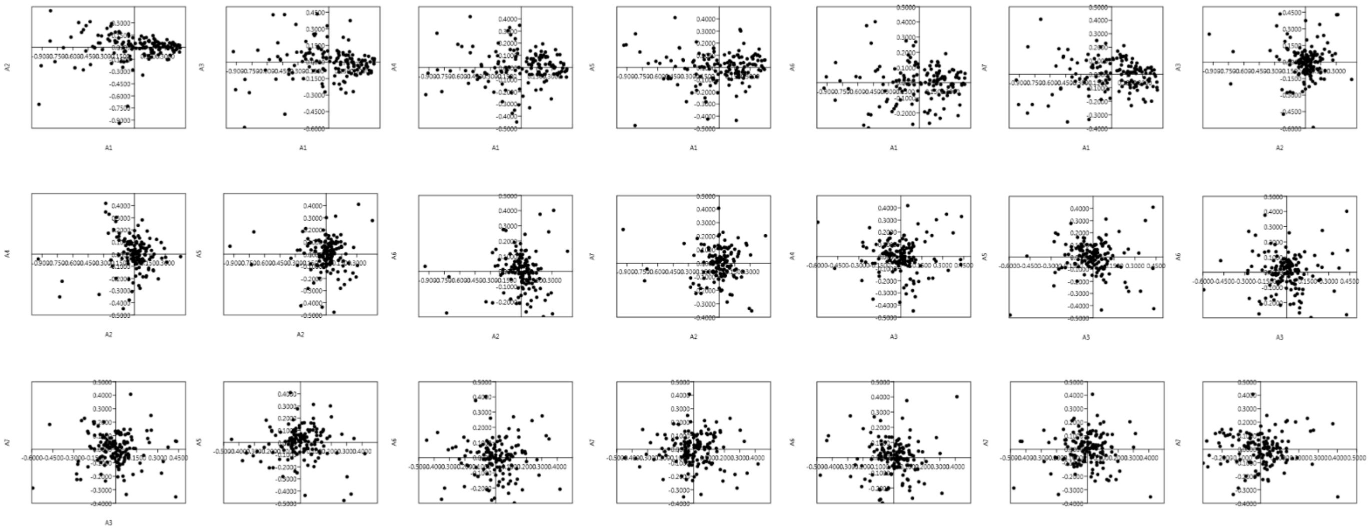

MH320550 (MCV 2)

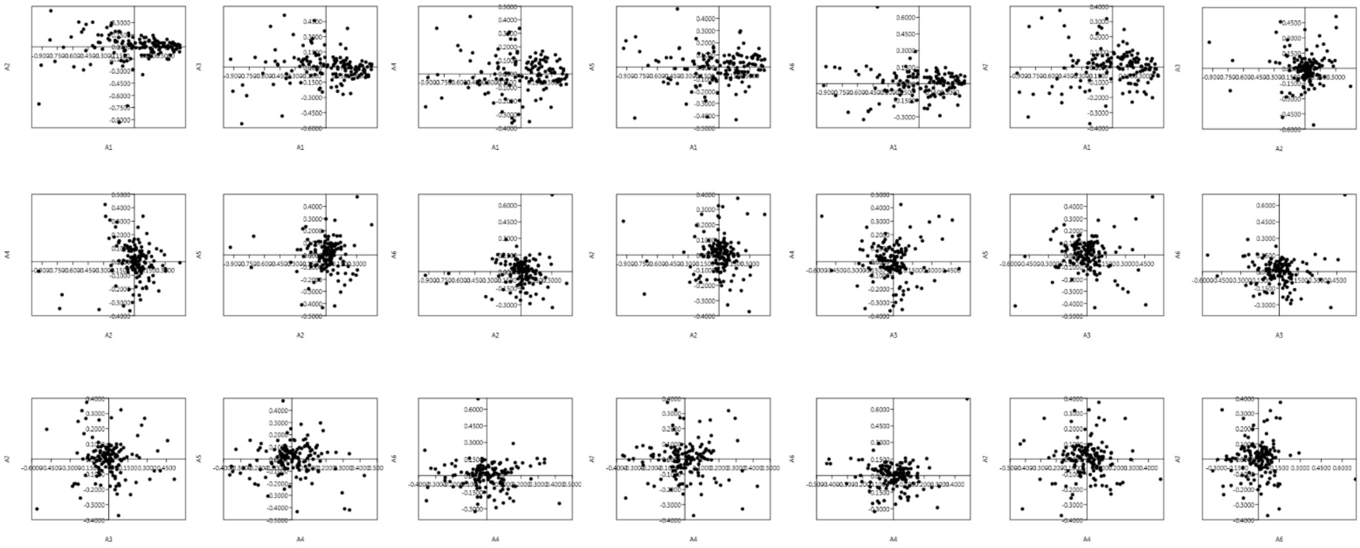

Figure S7. Coding sequences of MH320551 and MH320550.

MH320556 (MCV 2)

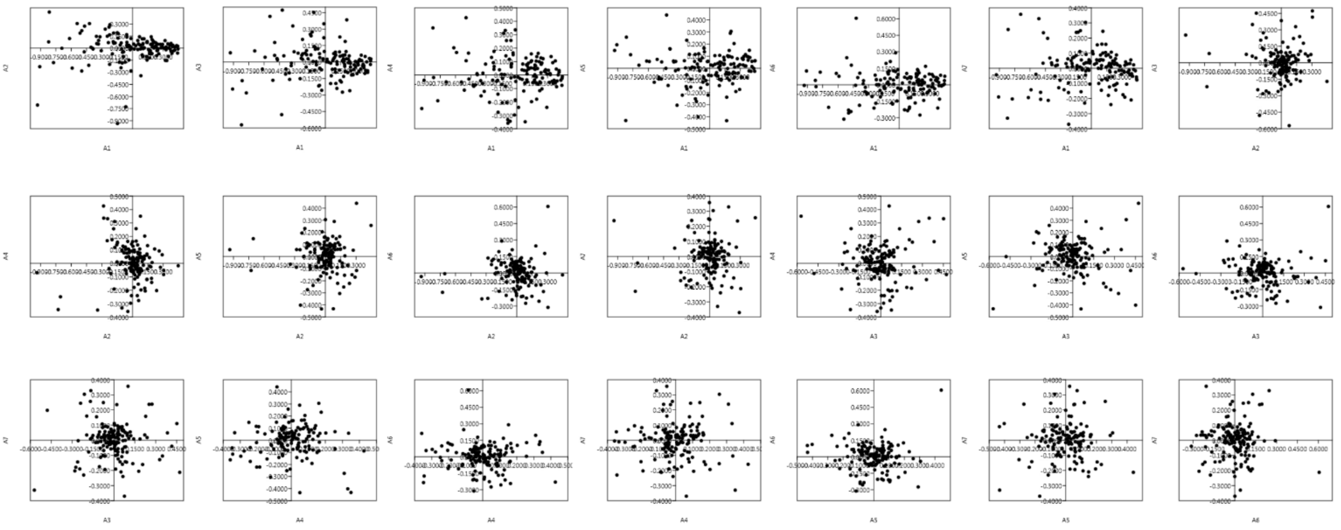

KY040274 (MCV 2)

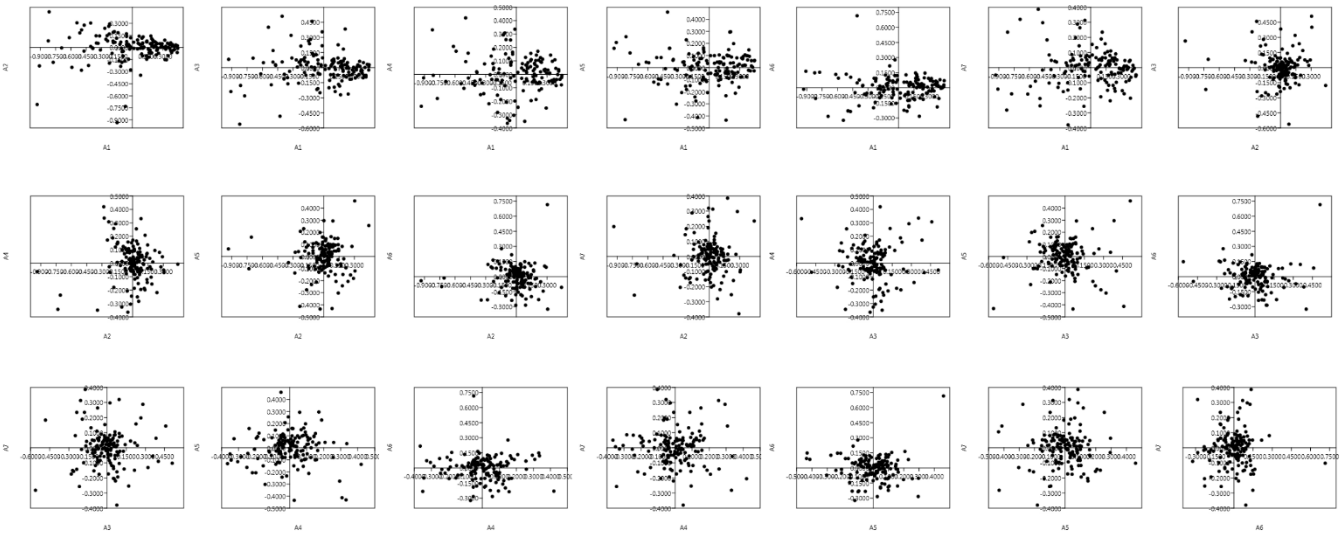

Figure S8. Coding sequences of MH320556 and KY040274.
